# Tsunamigenic potential of crustal faults and subduction zones in the Mediterranean

**DOI:** 10.1038/s41598-019-40740-1

**Published:** 2019-03-13

**Authors:** Patrizio Petricca, Andrey Y. Babeyko

**Affiliations:** 1 GFZ German Research Centre for Geosciences, Potsdam, Germany; 2 Present Address: Dipartimento di Scienze della Terra, Università Sapienza, Rome, Italy

## Abstract

We compiled a database and systematically evaluated tsunamigenic potential of all up-to-date known crustal fault systems and subduction zones in the entire Mediterranean region that has experienced several catastrophic tsunamis in historical times. The task is accomplished by means of numerical modeling of tsunami generation and propagation. We have systematically simulated all representative ruptures populating known crustal faults and subduction interfaces with magnitudes ranging from 6.1 up to expected Mw_max_. Maximum tsunami heights calculated everywhere along the coasts allowed us to classify the sources in terms of their tsunamigenic potential and to estimate their minimum tsunamigenic magnitude. Almost every source in the Mediterranean, starting from Mw = 6.5, is capable to produce local tsunami at the advisory level (wave height >20 cm and ≤50 cm). In respect to the watch level (wave height >50 cm) larger magnitudes are needed (M_w_ ≥ 6.9). Faults behave more heterogeneously in the context of far field early warning. De-aggregation of the database at any selected coastal location can reveal relevant sources of tsunami hazard for this location. Our compilation blueprints methodology that, if completed with source recurrence rates and site-specific amplification factors, can be considered as a backbone for development of optimal early warning strategies by Mediterranean tsunami warning providers.

## Introduction

Despite the fact that tsunamis in the Mediterranean region are not so often compared to active subduction zone settings as for Japan, Indonesia or Chile^1^, their socio-economic impact is large due to high coastal population density and infrastructure. The Mediterranean region has experienced several catastrophic tsunamis in Historical times^2^. The most destructive and well recognized events produced by tectonic sources^3^ occurred in AD 365 and 1303 (Crete), 1783 and 1908 (Messina Strait), 373 B.C. and 1748 (Gulf of Corinth). More recently, destructive tsunamis were recorded in 1956, near the Greek Island of Amorgos^4^ and in 2003 off-shore Algeria, near the city of Boumerdes^5^. Foregrounded after the tragic Sumatra tsunami on 2004 and under the coordination of the Intergovernmental Oceanographic Commission of UNESCO, Mediterranean countries are currently in the process of building their national Tsunami Early Warning Systems.

In planning and building of a Tsunami Early Warning System (TEWS), the first step is the assessment of the tsunami hazard. This analysis is usually been done by means of numerical simulations based on a set of tsunamigenic sources of different completeness. In the Mediterranean region, tsunami hazard assessments so far were performed in two different ways: (1) studies based on individual credible worst case scenarios^6–9^ or (2) probabilistic tsunami hazard assessment based on the concept of area sources^3^. Credible worst case scenarios provide the highest degree of detail; however, individual scenarios are inherently restricted to limited source variability, in particular, to prominent historical events or cases with largest expected magnitudes. Vast majority of possible tsunamigenic cases remain out of the scope of such studies. In contrast, the first probabilistic tsunami hazard assessment for the Mediterranean region^3^ considered the full range of magnitudes and extensive geographical coverage but the concept of area sources smeared-off seismicity from individual faults over broad geographical domains. Recently^10^, the probabilistic concept has been applied to the subduction type earthquakes along the Western Hellenic Arc thus moving conceptually from area sources to actual tectonic structures.

In the present study, we follow the deterministic approach but strongly increase the amount of analyzed sources – in fact, we present the first analysis of all up-to-now known real faults in the Mediterranean and provide a systematic evaluation of their tsunamigenic potential. Earlier, Necmioglu & Özel^11^ published an extensive deterministic analysis covering a wide range of magnitudes and hypocenters in the Eastern Mediterranean. In contrast to their study, which employs the concept of area sources, we settle ruptures along the mapped fault structures.

**Figure 1:**
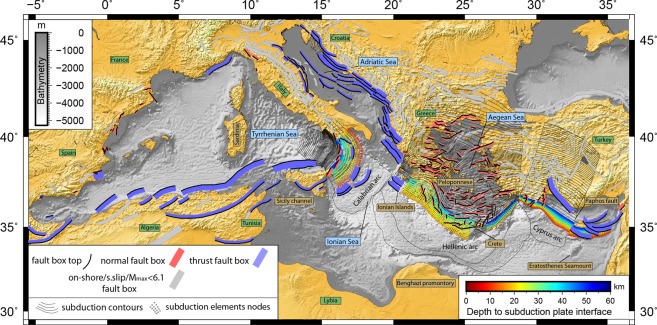
Composite sources (CSS) and subduction interfaces (SUBD). Data from European Database of Seismogenic Sources^12^ and DISS 3.2.1^13^. Blue (thrust) and red (normal) boxes are sources selected to be investigated. Dots (colors according to depth) represent nodes of the subduction seismogenic interfaces. Subduction contours are drawn every 10 km. Figure produced with the GMT 5.4.4 (http://gmt.soest.hawaii.edu/home).

The starting point for our study is the outcomes from previous EU-Projects collected in the European Database of Seismogenic Faults^12^ (EDSF) and integrated by the Database of Individual Seismogenic Sources^13^ (DISS v.3.2.1; Fig. 1). It is important to note that, despite existing disagreement between individual experts regarding the completeness and correctness of description of various faults and fault zones within Mediterranean, the above datasets can be considered as the only *consensus* database compiled by community of experts. We take these products ‘as it is’, without attempting to improve it in accord to individual suggestions, and hope that latter would finally result in an updated community consensus database to be used for the future studies.

From the EDSF and DISS datasets, we first identified and selected a sub-set of sources capable to trigger tsunamis in the study area. Further, we modeled ruptures and corresponding tsunami propagation scenarios considering all possible individual ruptures described by geometric and kinematic parameters. Individual sources were spatially distributed within fault structures and additionally ranged in magnitudes from Mw 6.1 to the maximum value assigned to each source. In total, we have simulated ca. 20.000 individual scenarios of tsunami generation and propagation. Maximum wave heights at coastal positions along the whole Mediterranean were recorded and then classified according to the warning level thresholds approved by IOC/UNESCO for the North East Atlantic and Mediterranean Tsunami Warning System (NEAMTWS). The resulting dataset allowed us to classify all known real faults in terms of their tsunamigenic potential, as well as to estimate their minimum tsunamigenic magnitude, which could be further utilized in the NEAMTWS.

## Definition and Parameterization of Sources

Two principal types of active tectonic structures are described in the EDSF^12^ and DISS v.3.2.1^13^, namely composite seismogenic sources (CSS) and subduction interfaces (SUBD)^14^. Source definition and our selection workflow are described in the following sections. Please also refer to the *Methods* section below for the detailed description of CSS and SUBD sources.

### Composite Seismogenic Sources (CSS)

A Composite Seismogenic Source (CSS) is a structure that represents a collection of potential individual sources that can produce earthquakes within a well-defined range of variation, in terms of rupture geometry and kinematic parameters^15^. In more practical way, CSS is a geo-referenced and fully-parameterized polygon with homogenous tectonic and geometric characteristics.

Not all the seismogenic sources proposed in the EDSF are believed to be capable of triggering tsunamis. For example, on-shore dip-slip and all strike slip CSS were excluded from the study (grey polygons in Fig. 1). To be more precise, we retained only off-shore thrust and normal faults having at least one point of their surface projection located within 20 km inland-buffer (red and blue polygons in Fig. 1) capable to trigger at least a 6.1 Mw.

From historical events it is known that strike-slip faults may also contribute to the tsunami generation^16^. This contribution is, however, attributed not to the vertical co-seismic surface deformation which is small in amplitude in case of strike-slip ruptures, but rather to secondary effects like horizontal translation of steep-slope topography or occasional earthquake-triggered landslides^16,17^. Since in this study we follow the most common concept for modeling of earthquake generated tsunamis, which is based on residual vertical deformation of the elastic half-space in respond to the finite subsurface dislocation^18^, we exclude these poorly constrained secondary factors from our study.

After applying this selection procedure we came out with 275 CSS source zones (out of a total of 1142 in the original databases) capable to generate notable tsunamis (Fig. 1) classified as:

- 187 normal (rake 270 ± 45) CSS (133 off-shore + 54 on-shore);
- 88 thrust (rake 90 ± 45) CSS (67 off-shore + 21 on-shore).

### Subduction interfaces (SUBD)

The Subduction source (SUBD) is a simplified representation of the plates interface at convergent boundaries based on geological and geophysical data^14^. In the original EDSF format, plate interfaces are presented by a set of depth contour lines depicting geometries of subducted slabs. Similarly to CSS, sources related to a subduction plate interface are characterized by geometric (strike, dip, depth) and behavior (rake, slip rate, seismic coupling and maximum earthquake magnitude) parameters.

Three subduction systems in the Mediterranean are described in the database: Calabrian, Hellenic and Cyprus arcs. Since we are interested only in potentially tsunamigenic ruptures along the subduction interfaces, we have limited subduction geometries to their shallow, seismogenic parts (depth shallower than 60 km). According to strategies adopted in previous works^10^, we divided the subducting slabs in along-strike sections to minimize parameters’ variability. The obtained geometries represent the projection of the subduction (seismogenic) interfaces on the Earth’s surface and are (moving from W to E): the Calabrian, the Hellenic-West, the Hellenic-East, the Cyprus-West and the Cyprus-East arc systems (Fig. 1). Calabrian arc unfolds for about 300 km with homogeneous parameters and smooth geometry between the Tyrrhenian Sea (to the west) and the Ionian Sea (to the east), hence considered as a single interface. The Hellenic-West geometry is based on a previously one proposed by^10^ and spreads between the Ionian Islands and Crete. Variation of the strike and dip of the subducting plate East of Crete, in regard to the western part of the arc, depicts the Hellenic-East interface. In the Cyprus arc, the continuity of the subduction front is interrupted west of Cyprus by the dextral strike-slip Paphos Fault^19^. This fault effectively marks the boundary between the oceanic domain subducting to the west and the collision domain to the east. This led us to consider the two distinct geometries for the Cyprus arc system (Cyprus-West and Cyprus-East).

### Evaluation method

We populate each CSS and SUBD source zone with a set of uniform-slip ruptures by varying their magnitudes from 6.1 to the specific source M_max_ and by moving their hypocenters within the geometrical limits of hosting composite source structure. For each rupture we compute tsunami propagation scenario (see *Methods* section below) and record maximum wave heights at more than 20.000 points of interest (POIs) evenly distributed along the 20 meter isobath with spacing of ~2 km.

As we aim to evaluate the tsunamigenic potential of individual seismic sources, we focus on classifying the input sources database rather than the tsunami effects at target points. In the tsunami glossary of the ITIC (International Tsunami Information Center) the word ‘tsunamigenic’ is simply defined as “capable of generating a tsunami”. To be more rigorous, here we define a source ‘tsunamigenic’ if capable of producing tsunami wave height over a given threshold at selected coastal points. Moreover, we rank a source as ‘tsunamigenic’ or not based not on any single POI but instead, at a group of POIs, thus, additionally introducing another threshold – “count threshold”. Such group evaluation plays a role of filtering of possible outliers. After testing different count thresholds (see Supplementary Information), we accepted a value of 10 POIs which corresponds to coastal segments of approximately 20 km length.

Our primary goal is to estimate the *minimum tsunamigenic magnitude* Mw_min_ which we define as the smallest magnitude of any rupture within CSS or SUBD source zone capable to trigger tsunami wave heights over the given threshold at least at 10 POIs. To evaluate Mw_min_ for each source zone, we start from the corresponding source M_max_ and proceed in 0.1 (CSS) or 0.2 (SUBD) magnitude steps down as long as the above ‘tsunamigenic’ criterion remains fulfilled.

Finally, rather performing simple binary classification of sources as “tsunamigenic?: yes or no”, we propose the following system of classification:

1. Mw_min_ triggering tsunami wave heights over the *advisory* level (i.e., WH > 20 cm and ≤50 cm) independent from source-to-POI distance (advisory-tsunamigenic);
2. Mw_min_ triggering tsunami wave heights over the *watch* level (i.e., WH > 50 cm) independent from source-to-POI distance (watch-tsunamigenic);
3. Mw_min_ triggering tsunami wave heights over the *advisory* level (i.e., WH > 20 cm and ≤50 cm) at *regional* distances (i.e., farther than 100 km from source; regional-advisory-tsunamigenic);
4. Mw_min_ triggering tsunami wave heights over the *watch* level (i.e., WH > 50 cm) at *regional* distances (i.e., farther than 100 km from source; regional-watch-tsunamigenic).

The above mentioned *advisory* and *watch* thresholds correspond to the wave height values that are adopted by IOC/UNESCO in the NEAMTWS for the related warning levels^20^, for which we have decided to use equality at the upper limits.

In light of these assumptions, Mw_min_ represents the minimum magnitude capable of triggering a tsunami that impacts group of POIs with wave heights over the *advisory/watch* level at *local/regional* distance. Important note: it is well accepted that the tsunami runup may strongly vary along the coast in accord with small-scale irregularities in shallow bathymetry. Hence, by accepting the group evaluation criterion (i.e., counting of at least 10 POIs above a given threshold), we tend to provide a rather *conservative* estimate of Mw_min_. There always be individual coastal spots with “individually” smaller Mw_min_. In this respect, our analysis follows the tsunami forecasting by the Japan Meteorological Agency (JMA) always stating the possibility of locally higher runups as predicted for a coastal segment.

## Results

In this section we will present evaluation results of the minimum tsunamigenic magnitudes Mw_min_ for the Mediterranean sources. Since these results, by fact, are of predictive character, they can be compared against known historical events. This will be done later, in Discussion section.

### Tsunamigenic potential of individual sources

**Figure 2:**
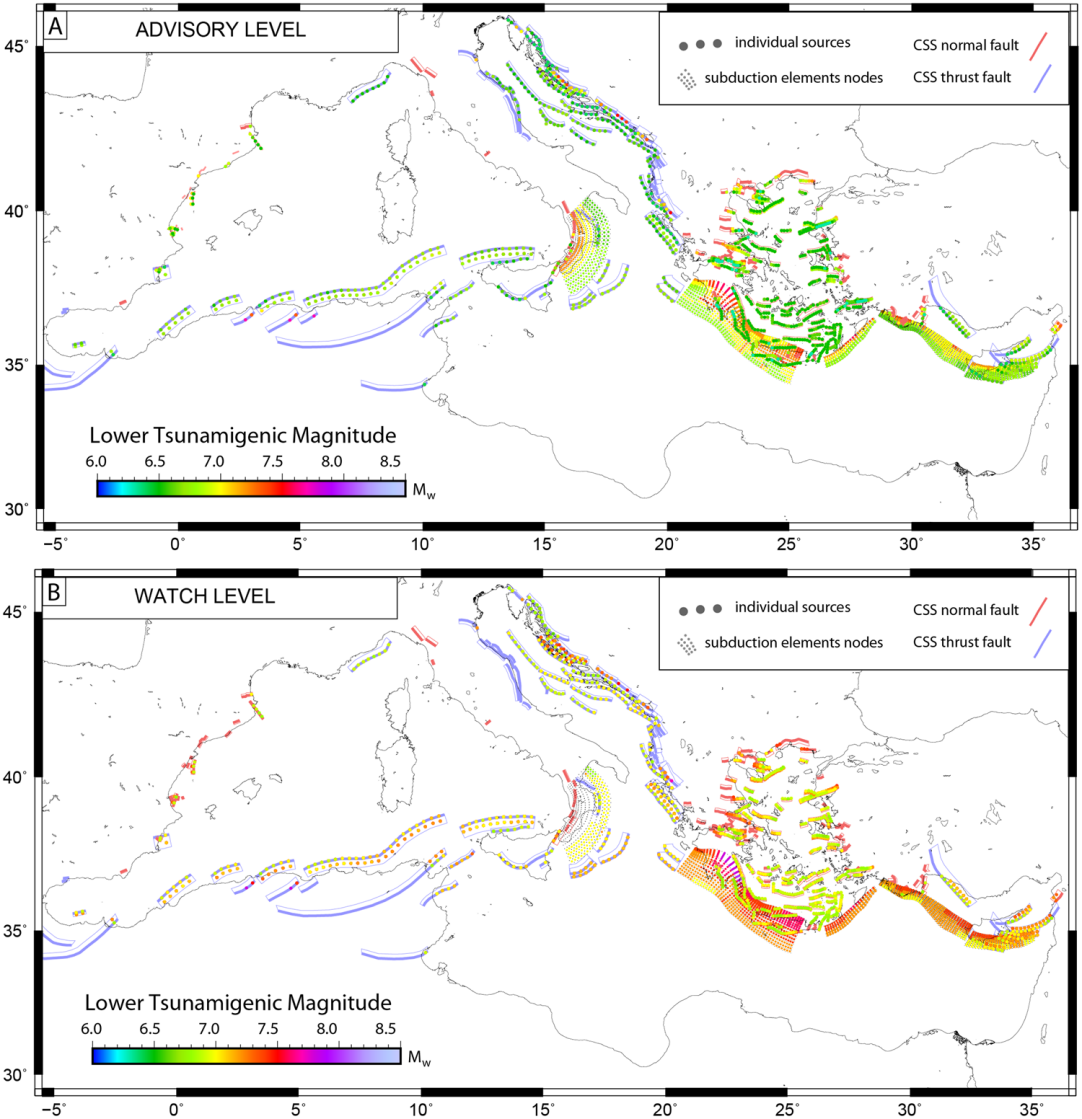
Minimum tsunamigenic magnitude (Mw_min_) of individual sources. Mw_min_ necessary to produce advisory (wave height >0.2 m and ≤0.5 m; (**A**) or watch (wave height >0.5 m; (**B**) alerts along the Mediterranean coasts are provided for CSS and SUBD composite sources. Figure produced with the GMT 5.4.4 (http://gmt.soest.hawaii.edu/home).

**Figure 3:**
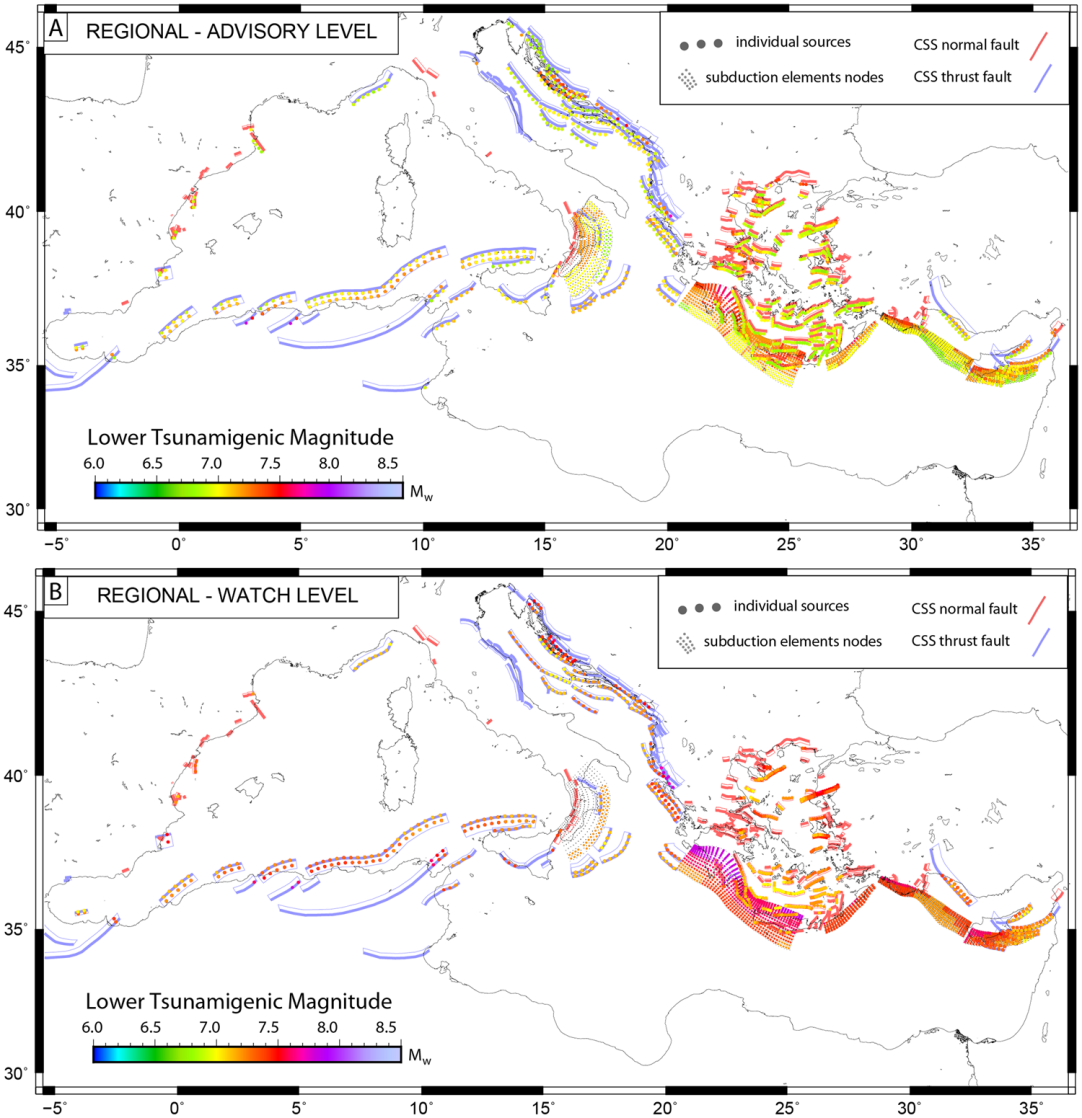
Minimum tsunamigenic magnitude (Mwmin) of individual sources at regional distance. Mw_min_ necessary to produce advisory (wave height >0.2 m and ≤0.5 m; (**A**) or watch (wave height >0.5 m; (**B**) alerts along the Mediterranean coasts located farther than 100 km from epicenters (regional tsunamis) are provided for CSS and SUBD composite sources. Figure produced with the GMT 5.4.4 (http://gmt.soest.hawaii.edu/home).

In Figs 2 and 3 we present the results in terms of tsunamigenic potential as a characteristic of individual sources. The minimum magnitude capable of producing tsunami at least at 10 POIs is presented under the four classification criterion introduced above: 1) advisory; 2) watch; 3) advisory-regional; 4) watch-regional. Note, since there can be several individual sources with different magnitudes at each position within a composite source or subduction interface structure, color at any individual position (dots on Figs 2 and 3) corresponds to the minimum tsunamigenic magnitude at this position.

Looking at the ‘advisory’ level (0.2 m < WH < =0.5 m; upper panel - Fig. 2) clearly emerges that earthquakes with relatively low magnitudes are already capable to trigger tsunami alerts. Magnitudes from CSS compilation span mainly in the Mw 6.5–7.4 range, with general increase depending on distance to the closest coast and hypocenter depth. As an example, the composite source stretching off-shore Tunisia and Sardinia clearly requires higher magnitudes when moving away from shorelines (increasing ≈ 0.3 in magnitude from eastern to western tips) and also comparing the (northern) shallow sources with their (southern) corresponding at higher depth (note the colors changing from green to light-green/yellow tones). Consistent values result from all subduction interfaces, all showing values over Mw 6.6. As expected, higher magnitudes are needed to trigger tsunamis for sources lying partly on-shore (e.g., around the Aegean Sea, entering the north Africa coasts or for the Croatian sources in the Adriatic Sea), but never exceeding Mw 7.0–7.4.

Moving to the ‘watch’ warning level perspective (WH > 0.5 m; lower panel - Fig. 2), most of the ruptures show a magnitude increase, now spanning in the Mw 6.7–7.9 range. Minimum magnitudes for subduction zones increase to Mw > 6.8 for the Calabrian arc, Mw > 7.0, for the West Hellenic, West and East Cyprus arcs and Mw > 7.2 for the East Hellenic arc.

At regional distances (i.e., more than 100 km between source and target points), minimum magnitudes necessary to trigger ‘advisory’ alerts remain generally under Mw 7.0 for the enclosed seas (Adriatic and Aegean) and Mw 7.4 moving inland, while for the remaining regions they increase over Mw 6.9 (upper panel - Fig. 3). At the ‘regional-watch’ level (lower panel - Fig. 3), minimum tsunamigenic magnitudes show the largest inhomogeneity: Mw ranges from 7.0 to 7.9. The lowest values are observed around the Adriatic and Aegean Seas and for the Calabrian arc, while the highest for the deeper part of the subduction zone of the West Hellenic Arc (Mw > 8.0).

Note the un-colored portion of the Calabrian Arc at the lower panel of Fig. 3, as well as the fact that in the Adriatic sources are missing (compare upper and lower panels of Fig. 3). That is because individual sources at these regions were not able to trigger the corresponding threshold level (here- ‘regional watch’) even at their highest possible magnitudes (e.g. the Calabrian Arc saturates at Mw > 7.2).

### De-aggregation at selected target sites

As application of our scenario database, we made de-aggregation in order to assess tsunamigenic potential of individual sources affecting selected test sites. Since coastal communities have specific geographical extent along the coastline, their representation with one single POI would be non-sufficient and prone to outliers. To get more stable results, we do average tsunami wave heights along all the POIs belonging to a community (see Supplementary Information for illustration of POI distribution for test sites – Figure S4). Remembering that POIs are just coastal nodes of the 1 arc minute GEBCO bathymetric grid (i.e., the average spacing between POIs is about 2 km), we may expect that the POI-averaged wave height will be rather a conservative estimate for a test site as there will be always coastal points with larger tsunami amplitudes. This, in turn, means that our analysis tends to overestimate the minimum tsunamigenic magnitude regarding particular test site.

**Figure 4:**
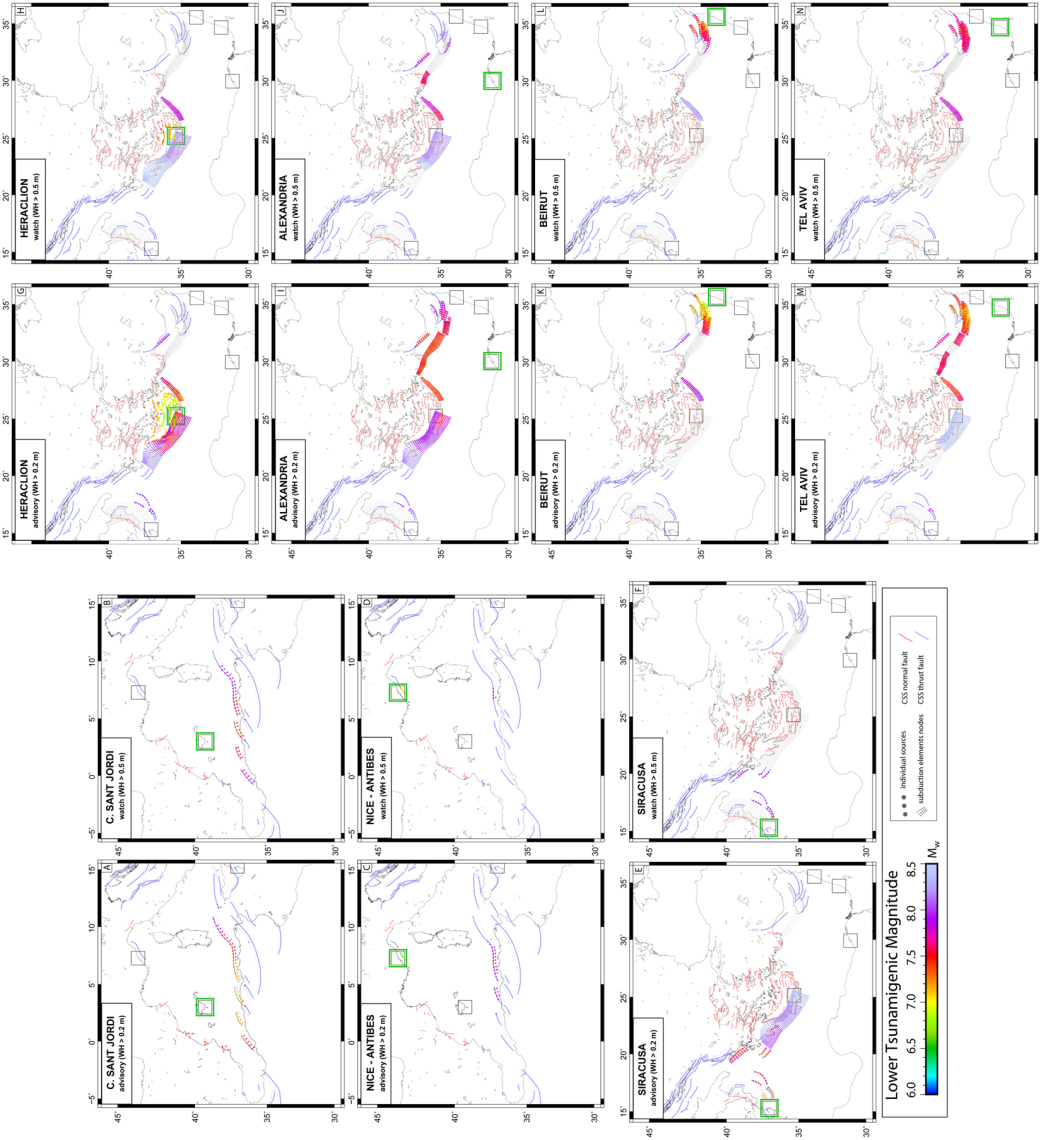
Minimum tsunamigenic magnitude (Mw_min_) of individual sources necessary to produce advisory (left) or watch (right) alerts at selected test sites (Black boxes). Target test sites are highlighted by green boxes. Figure produced with the GMT 5.4.4 (http://gmt.soest.hawaii.edu/home).

Figure 4 presents de-aggregation results in both ‘advisory’ (left panels) and ‘watch’ (right panels) warning levels for sites considered representative for western, central and eastern Mediterranean basin. Namely: Colonia Sant Jordi (Mallorca Island - Spain) and Nice (South France) in Western Mediterranean, Siracusa (Sicily Island - Italy) in the Ionian Sea; Heraklion (Crete Island - Greece) in the Aegean Sea, Alexandria (Egypt), Beirut (Lebanon) and Tel Aviv (Israel) in Eastern Mediterranean.

Results for Colonia Sant Jordi show that this community is potentially affected by events originating at the North African sources and few seismogenic sources offshore Spain. North Sicily sources produce advisory alert (wave heights >20 cm; ≤50 cm) considering high magnitude events (Mw > 8.0; Fig. 4A), while no watch alert (wave heights >50 cm) is expected (Fig. 4B). Advisory alert is predicted for Mw between 7.0 and 8.2 while watch level requires Mw between 7.3 and 8.2 depending on distance and depth of the source (note that 8.2 is the maximum expected magnitude of those seismogenic sources).

Nice-Antibes is affected by events along the same North African composite sources, as well as by more local source stretching in front of its coast (Fig. 4C,D). If we consider the African sources, Mw in the range of 7.3–8.2 and 7.7–8.2 can trigger advisory and watch level alerts respectively. The source close to Nice requires smaller magnitudes starting from Mw 6.6 for advisory and from 7.0 for watch level.

Advisory alert in Siracusa (Fig. 4E) can be triggered by ruptures along the nearby sources offshore Calabria with Mw > 7.1, as well as by larger magnitude earthquakes offshore western Greece. It is interesting that West Hellenic Arc must produce at least Mw 8 earthquake to trigger advisory level in Siracusa. This is probably explained by the two factors: (1) source directivity towards the African coast, i.e., tsunami wave has to be first reflected from this coast before it reaches Sicily and (2) relatively deep sources along the West Hellenic Arc: the depth of the upper seismogenic zone is 18 km, according to this subduction-structure description. Watch level alert (Fig. 4F) is recorded at Siracusa coasts for relatively high magnitudes (Mw > 7.5) produced at nearby off-shore Calabria and West-Greece sources.

Tsunami hazard for Heraklion is produced principally by sources at local distances given the close presence of the two branches of Hellenic subduction zone (west and east) and the numerous sources existing in the Aegean Sea (Fig. 4G,H). Advisory-level magnitudes span from 7.1 to 8.2 for the two arc branches while shallower crustal sources in the Aegean, just off the coast of Heraklion, need much smaller magnitudes (from 6.5 to 7.4) to trigger alerts in this city. Regional events are also predicted to Heraklion considering Mw > 7.8 produced at sources nearby Calabria and South Turkey-Cyprus coasts (Fig. 4G). Watch level alerts are produced at Heraklion site in a very local domain (Fig. 4H). Hellenic subduction West and East branches are the more effective warning sources for the site, because of the geographic location and capability of high magnitude events (up to 8.4). Although they are shallow, only crustal sources in the Aegean Sea very close to Heraklion produce watch level at site’s coasts, because of the lower magnitudes of the earthquakes (Mw_max_ 7.3) produced here by normal faults.

Due to its position, Alexandria is prone to effects of all East-Mediterranean sources. Off-shore Calabria sources, Hellenic and Cyprus Arcs and sources south of Turkey produce advisory alerts for magnitudes in the range of 7.4–8.4 (Fig. 4I). Higher magnitudes (Mw > 7.6) required to produce watch alert are due to ruptures belonging to closer (with respect to Alexandria) tsunamigenic sources (Fig. 4J).

Sources at local distances (i.e., crustal sources off-shore Turkey, Cyprus and the East-Cyprus Arc) produce alerts to Beirut (Fig. 4K,L) and Tel Aviv (Fig. 4M,N), that represent the easternmost sites selected to apply our database outcomes. In the advisory level, high magnitudes (Mw > 7.9) in the Ionian Sea off-shore Calabria and along subduction interfaces (excluding Calabrian Arc) must be considered to Tel Aviv (Fig. 4M). East branch of the Hellenic Arc produce some level alert to Beirut (Fig. 4K,L) and watch level alert to Tel Aviv (Fig. 4L). It is worth mentioning that tsunami wave originated at crustal sources off-shore the Calabria have sufficient energy and the optimal basin physiography to cross the whole east-Mediterranean and pone advisory threat to the city of Tel Aviv (Fig. 4M).

## Discussion

Besides the Probabilistic Tsunami Hazard Analysis^3,10^ (PTHA), deterministic studies based on few selected scenarios were widely used to assess historical events as well as possible tsunami hazard for the Mediterranean^6–8,21,22^. Such an approach, also called as Worst-case Credible Tsunami Scenario Analysis^9^, aims to present the effect of extreme events at a specified location from a limited number of sources. In our study, we extend this approach by analyzing all possible events due to all known tectonic sources producing threats along the whole Mediterranean domain. By analyzing the broad range of magnitudes - from maximum possible down to minimum tsunamigenic - we primarily look at the tsunamigenic potential of the tectonic sources rather than concentrating on specified coastal locations. This approach gives us a comprehensive view over the whole Mediterranean, but, on another hand, makes it not straightforward to compare our findings with previous studies about worst credible cases.

Our first result is that most of the Mediterranean coasts are prone to potential tsunami considering known tsunamigenic sources (Fig. 2) for relative low magnitudes. Every source in the Mediterranean is capable to produce local tsunami at the advisory level (wave height >20 cm; <=50 cm) starting from Mw 6.5 (Fig. 2A). Larger magnitudes (around Mw 6.9) are needed to trigger watch levels (wave height >50 cm) even at the nearby coasts (Fig. 2B). Regional warning (source to coast distance >100 km) at advisory level is produced with magnitudes as low as Mw 6.9 (Adriatic See, Aegean), while Mw > 7.2 are necessary from sources in south-western Mediterranean, Aegean Sea as well as all subduction sources at watch level (Fig. 3). These magnitudes are lower than the values used for the worst-case/most-credible tsunami scenarios evaluated in previous studies (e.g., for north Algeria-Tunisia and Hellenic arc^7^; in the Adriatic^8^; for the Ionian^23^; for Cyprus Arc^9^), highlighting the importance of analyzing the entire spectrum of magnitudes associated with each individual source.

**Figure 5:**
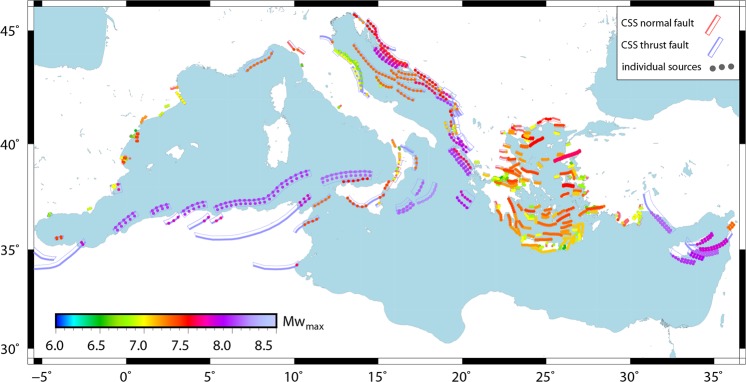
Composite Seismic Sources (CSS) in the Mediterranean region. Dots represent geometric centers of individual sources colored according to the maximum expected magnitude assigned to the corresponding CSS. At each point magnitude ranging from Mw 6.1 to Mwmax with increments of 0.1 was implemented (see methods section). Figure produced with the GMT 5.4.4 (http://gmt.soest.hawaii.edu/home).

The largest number of advisory/watch tsunami occurrences is close to the subduction interfaces and along north-African and Italian coasts (Figure S1). Gaps and peaks of occurrences characterize the East-Tunisia-Libya, South Turkey, Spain and France coasts, mostly following the distribution of known faults (cf. Fig. 5), as well as their different values of Mw_max_. Similar distribution emerges for PTHA maps of Mediterranean considering 5000 years return period^3^.

Subduction interfaces stretch in the Eastern Mediterranean and, consequently, tsunamis triggered by them show propagations paths confined in that area (Figure S2). An important outcome of our compilation is that tsunamis originated at subduction interfaces (i.e. in the East-Mediterranean basin) do not overpass the Sicily channel connecting the East- and the West Mediterranean, or at least produce wave heights at West-Mediterranean coasts under 20 cm (i.e., below the advisory level). These results support the previous findings by^7,24^ and confirm the idea that the tsunami perspective in the West-Mediterranean is controlled by the distribution of known local crustal seismogenic sources, while the high tsunamigenic potential of subduction zones significantly contributes to the tsunami hazard in the East-Mediterranean.

Recently, an extensive tsunami simulation databank for the East-Mediterranean was published^11^. We can use their results to compare/validate our current study. Although, different methodologies do not make possible direct one-to-one comparison (e.g. we used individual faults and subduction zone structures whereas they^11^ distributed epicenters evenly by longitude and latitude, and our coastal points-of-interest are spaced much denser), comparing the two scenario databases in respect to their predicted minimum tsunamigenic magnitudes (Mw_min_) shows very similar distribution necessary to trigger advisory or regional watch alerts (i.e., the two end-members of our tsunamigenic classification; see Figure S3 in Supplementary Information). Differences emerge at the locations of the subduction zones (Hellenic and Cyprus Arcs). In fact, our compilation provides along the subduction plate interfaces greater Mw_min_ values with respect to those given in^11^ (up to 7.5 vs 7.0 in the deepest part of the West Hellenic Arc). Differences emerge since our hypocenters deepen along the subduction zone geometry starting from 18 km (Hellenic Arc) and 10 km (Cyprus Arc) depth deepening gradually down to 60 km, while depths in the compilation from^11^ are fixed at 5 km (shallow sources) and 40 km (deep sources). This show how shallow sources (depth = 5 km) are more effective in tsunamigenesis with respect to deeper sources (depth ≥10 km) along the subduction structures, thus requiring much lower values of Mw_min_ (Figs 2, 3 and S3). Again, notice that for our compilation in the places where crustal sources overlie subduction interfaces (region of West Hellenic and East Cyprus Arc) these crustal faults are colored according to lower magnitudes. Similar conclusions emerge from comparison with previous works. If we discard shallow sources from^11^ and let only deep sources (depth >40 km), resulting magnitudes in the arc regions will be very similar to our study (See Figure S3 in Supplementary Information).

As in any modeling study, we also had to accept values for numerous model parameters regarding both source and propagation modeling. To evaluate sensitivity of our final results to most critical assumptions, we conducted a series of sensitivity checks. In particular, we tested sensitivity of Mw_min_ to the placement of individual sources within their hosting CSS structure; to the POIs count threshold and to the positioning of the off-shore POIs. Please refer to the Supplementary Information Figures S5 to S7.

Through site de-aggregation of results we identified the main faults associated to the tsunami hazard at specific sites (cities) along the Mediterranean. Such an analysis may, first of all, be useful for planning of local hazard mitigation and early warning because it identifies the potential sources that have to be considered at specific locations. De-aggregation analysis also highlights characteristics that favor a source to become tsunamigenic. In this respect, primary factor is the source-to-site distance. Minimum magnitudes required to alert a site systematically increase by moving from local to regional sources (in our classification). See, e.g., Colonia Sant Jordi and Nice-Antibes (Fig. 4A–D). On another hand, the case of Siracusa (Fig. 4E,F) also shows the role played by the depth of the source. Here, the West Hellenic Arc must produce at least Mw 8.0 earthquake to trigger advisory level which is explained by relatively deep sources along the West Hellenic Arc (>18 km in our compilation).

By comparing predictions from our databank with historical cases we can better assess limitations of our current study as well as formulate recommendations for future improvements. For example, ‘watch alert’ (WH > 50 cm) at Colonia Sant Jordi will be attributed to M > 7.2 earthquakes off-shore North-African coast (Fig. 4A). On another hand, a smaller Mw 6.9 2003 Boumerdes earthquake triggered 60 cm wave height within the Palma harbor located at about 40 km distance^25^. Earlier analysis of this event^26^ concluded that the significant wave amplification was generated by a resonance effect induced by Palma bay. (It is worth to note that, despite the bay resonance, authors also proposed to revise source mechanism of the Boumerdes tsunami.) The above example suggests that the site-specific assessment of hazard potential requires much higher bathymetry resolution. Since this is computationally often not feasible, we suggest to combine coarse-grid propagation simulations with site-specific amplification factors^27,28^.

Another historical case which may question our Mw_min_ results is the impact of the Crete AD365 event at the Sicilian coast. Whereas scenario dataset predicts that no ‘watch-alert’ in Siracusa can be triggered by the West Hellenic Arc (Fig. 4F), paleotsunami deposits in Eastern Sicily close to Siracusa may be associated with the AD365 event^29^. Literature analysis and additional modeling showed that, probably, it was the specific nature of the AD365 rupture which was not reflected by our source model of the Hellenic Arc filled with inter-slab events subjected to usual scaling relations. In particular, we were able to reproduce modeling results of ^7^ (including ~1.5 meter wave at Siracusa) using their source model of the AD365 event. However, this rupture was placed much shallower than the plate interface and had size and slip parameters not compatible with known scaling relations, actually representing an unusually high slip along the shallower crustal steep-dipping fault within the overriding plate, see also^30^. This use case demonstrates limitation of the input source databases accepted for the present study and the need to continue upgrading classification and quantification of potential Mediterranean sources. Another restriction can be assumption of the uniform slip. In present study, this assumption is a valuable approximation because the vast majority of sources have Mw_min_ below 7.5 (see Modeling approach - Source). However, site-specific analyses like that for Siracusa may operate with larger magnitudes corresponding to larger rupture dimensions. In this case, neglecting slip concentration may result in hazard underestimation^31^. Stochastic slip rupture modeling will cover the relevant uncertainty but, when coupled to propagation modeling, becomes computationally very challenging. A new approach based on surface tsunami Green’s functions^32^ may be opted for the future studies.

As a final remark, we note that current compilation does not integrate recurrent rates of seismic sources. The probabilistic aspect, if being included into the future studies, will definitely increase the value of such a compilation for the tsunami hazard assessment, mitigation and early warning in the Mediterranean.

## Conclusions

We present the methodology and the first evaluation of tsunamigenic potential of up-to-now known fault systems and subduction interfaces in the Mediterranean and propose a criterion for their classification. Generation of all possible ruptures and propagation of associated tsunamis allow us to define the minimum tsunamigenic magnitude Mw_min_, i.e. the lowest magnitude for each single source capable to trigger a tsunami at any alert level and at certain distance (advisory, watch, regional-advisory and regional-watch). Due to the accepted methodology, our metric provides rather a conservative estimate meaning that earthquakes of given Mw_min_ and location will definitely trigger a given alert-level; while there could be also smaller earthquakes that may locally (spot-wise) trigger even higher wave heights. Results show that Mw ≥ 6.5 ruptures occurring off-shore are energetic enough to produce tsunamis at the ‘advisory’ level (20 cm < h_max_ < 50 cm, with h = wave height) in almost all the Mediterranean Sea. Mw 6.9 is needed to trigger ‘watch’ (h_max_ > 50 cm) alert even at the nearby coasts. In the context of the ‘regional warning’ (source-to-site distance >100 km), these thresholds increase to Mw 6.9 for the ‘advisory’ level and to around Mw 7.2 for the ‘watch’ level. Minimum values are observed around the enclosed seas (Adriatic and Aegean), while the highest magnitudes are necessary for the deeper part of the subduction zone of the West Hellenic Arc (Mw > 8.0).

Coastal locations with the largest number of potential tsunami occurrences (counted as number of ruptures which could in principle affect each point of interest) are distributed close to the subduction interfaces and mostly follow the distribution of known crustal faults as well as faults with the higher values of Mw_max_. This means that the West Mediterranean suffers from tsunamis generated at crustal sources, whereas high tsunamigenic potential of subduction interfaces dominates in East Mediterranean, emphasizing the importance of improving the geological knowledge of a region in order to increases the confidence in tsunami warning assessment. As shown for selected test sites, the power of the presented methodology is the opportunity to extract threats for selected sites that, if extended with source recurrent rates and site-specific amplification factors, can be used for planning of optimal early warning and mitigation strategies by Mediterranean regional and national civil protection authorities and warning centers. Progress of this work crucially depends on quality and completeness of source classification and quantification in Mediterranean.

## Methods

We operate with two different formats describing source parameters. (1) A gridded source which is effectively a regular distribution of a number of individual faults within given planar structure (e.g., within a CSS). In this case, each point of the grid (Fig. 5) represents a center of a family of rectangular faults described by geometric parameters (depth, strike, dip, and rake) and magnitudes ranging from Mw 6.0 to the maximum expected for this particular CSS. (2) A subduction plate interface represented by a complex 3D-surface discretized by an ordered set of quadrilateral patches (complex plane-type) which can host multi-patch ruptures with magnitudes up to maximum expected. For definition of dip, strike and rake in the EDSF-database the reader is referred to^14^.

### Crustal sources (CSS) - Gridded-type input

Our compilation of grid source-type provides distribution of sources within crustal composite sources (CSS) of normal and reverse type (Fig. 5). Grid step is not homogeneous within CSS, but the distance along strike between any two consecutive points (i.e., between the centres of two consecutive individual sources) is always less than half of the typical rupture length of a Mw 6.5 earthquake (~12 km according to scaling laws^33^). This value of 12 km decreases to a minimum of 8 km where we search for higher resolutions (e.g. normal faults, characterized by lower expected magnitudes). Similar procedure was applied for distribution of individual sources along the dip of a source. If interface is wider than 12 km (i.e., typical rupture width of Mw 6.5 earthquakes^33^), a second row of individual sources was distributed at depth by simple shifting down the initial series of sources by 12 km. The maximum depth reached by CSS is 25 km, which makes our compilation complete in a way that individual sources fully cover all selected composite sources.

Worth remembering that the original maximum expected magnitude (Mw_max_) given in the EDSF^12^ is not uniformly determined for different CSS’s. Its value is usually based on the strongest past event occurred along that or adjacent structure, or on geological/geophysical evidences (e.g. length of mapped surface trace, fault size) coupled with magnitude scaling laws. In our opinion, approach based on largest past event may result in underestimation of maximum possible magnitudes, especially when the estimate is based on historical or instrumental events. For this reason, we decided to use Mw_max_ from the scaling laws considering the largest fault segment composing CSS^12^. The largest value from different scaling relations was used^33–37^.

### Subduction interfaces (SUBD) - Complex plane input

Subductions are complex tectonic features and most of tsunamigenic earthquakes occur along their seismogenic interfaces. Rupture geometry can affect the tsunamigenic potential^38^, making it necessary to properly model the fault. In order to give due weight to the geometry of the system, we specifically designed the input file for subductions. Subduction interfaces are collected as complex 3D-surfaces, each described by an ordered set of nodes, each node parameterized by coordinates and depth. In the plate interface description file, nodes are organized as isolines of depth. Starting from nodes, we build the interface and routinely define other geometric parameters (depth, dip, strike) for the enclosed quadrilateral sub-elements (‘patches’) composing the whole structure.

The upper isoline corresponds to the minimum seismogenic depth^15^; the bottom isoline is set to 50 km depth according to^39^. To populate SUBD interfaces with individual sources, we have evenly distributed ruptures of increasing magnitudes along the whole plate interface. Magnitudes were increased starting from Mw 6.6 to Mw_max_ with 0.2 step. For each magnitude, we filled the whole plate interface along its strike and dip with a sufficient number of individual uniform-slip ruptures. Rupture dimensions were estimated from the scaling relations of^33^. Rake was set to 90° (pure thrust). Statistics for individual subduction interfaces are summarized in Table 1.

**Table 1:** Statistics for subduction interfaces.

					n. of simulated ruptures (by M_w_)			
Subduction ID	min depth (km)	max depth (km)	Mw_max_	rake	6.6–7.0	7.2–7.6	7.8–8.2	8.4
Calabrian	10	50	7.2	90	301	69	—	—
Hellenic-West	18	51	8.4	90	433	149	36	4
Hellenic-East	18	51	8.4	90	208	39	9	1
Cyprus-West	10	50	7.6	90	308	114	—	—
Cyprus-East	10	50	7.6	90	269	86	—	—

### Modeling approach – Source

We use *RuptGen* tool to simulate initial conditions for tsunami propagation^40^. Co-seismic sea bottom deformation is computed following the analytical solution for a rectangular dislocation within the linear solid halfspace^17^. In case of individual faults (here- CSS sources), *RuptGen* implements the single fault model whereas in case of curved plate interfaces (here- SUBD sources), it first defines the ruptured part of a given plate interface and then distributes corresponding seismic moment along the patches of the ruptured area. In both cases, scaling relations^33^ are used to estimate fault dimension and amount of slip. In present study, slip was distributed uniformly. This simplification is, however, fully acceptable because our ‘target’-magnitudes, i.e., minimum tsunamigenic magnitudes, are generally lower than 7.5 (except for very deep sources, see Figs 2 and 3).

### Modelling approach – Tsunami

For each individual source, corresponding tsunami propagation scenario was computed using the *easyWave* propagation code (https://gitext.gfz-potsdam.de/geoperil/easyWave.git). *easyWave* follows the classical numerical algorithm by^41^ for simulation of linear long-wave propagation in spherical coordinates. For benchmarking and comparison of *easyWave* against other propagation codes see^42,43^. Tsunami propagation was computed on the GEBCO_08 (General Bathymetric Chart of the Oceans) bathymetric grid (http://www.gebco.net) resampled to 1 arc minute, and maximum sea level heights after four hours of propagation were recorded in each computational node along the vertical reflecting wall placed along the 20-meter isobaths. This type of boundary conditions is commonly used for propagation simulations without inundation: the full reflection converts whole kinematic energy into the wave height with amplification factor of up to 2 (in linear theory) and is being accepted as feasible approximation for coastal runup. Placing the sea-land boundary along the 20 meters isobaths allows exclusion of small-scale coastal bathymetric features like shallow bays with highly variable and irregular local runup. We consider each computational node at the reflecting wall as a point of interest (POI). Totally, we used 23236 POIs equally distributed (with ~2 km spacing) along the whole coast of the Mediterranean and internal seas.

Linear tsunami modeling at a coarse 1’ bathymetric grid cannot simulate coastal impact in details: realistic modeling at a local scale requires not only nonlinear numerical schemes able to simulate wetting/drying but also extremely fine bathymetric grids (down to 10 m resolution) coupled to digital terrain models with sub-meter vertical accuracy. Despite this note is fully approved, we argue that linear *easyWave* can still be accepted as an adequate tool within our modeling framework.

#### Computational argument

Modeling of hundreds of thousands of scenarios in the highest resolution is nowadays yet not possible. That is why regional studies with large number of scenarios (like PTHA) usually restrict tsunami propagation to the linear deep water domain (depth >20–50 m) where coarser grids and computationally inexpensive linear codes can be applied.

#### Resolution argument

1’ grid resolution corresponds to about 2 km distance between the computational nodes. As a result, in most places in the Mediterranean, deep-water domain comes out already few nodes off the coast; so that incident wave will not have enough space to perform non-linear transformations. Due to the same reason, we decided not to apply the Green’s law to the off-shore nodes as in some previous studies^3,11^. This law has to be applied to a free passing wave not affected by coastal reflections. This condition definitely is not applicable at a 1’ grid as “deep-water” nodes lie in the closest vicinity to reflecting coastlines. Instead, we decided to record the wave height directly at the land/water interface (placed at the 20 meter isobaths), where wave splashing already gives amplification factor up to 2.

#### Amplitude argument

Out ‘target’-amplitudes correspond to the ‘advisory’ and ‘watch’ levels which are 20 and 50 cm, respectively. Since these values are much smaller that the water depth even at the last water nodes, linear approximation becomes valid for our results on minimum tsunamigenic magnitude.

Of course, simulation on a coarse grid cannot account for local effects and, in general, will underestimate maximum coastal runup^44^. Our coastal wave heights should rather be considered as conservative estimates. In turn, thus derived minimum tsunamigenic magnitudes (Figs 4–5) should be considered as estimates from above.

## Supplementary information

Supporting material
