# Supplementary material for: Tsunamigenic potential of crustal faults and subduction zones in the Mediterranean
Source: Sci Rep. 2019 Mar 13;9:4326. doi: 10.1038/s41598-019-40740-1 (PMC6416261; doi:10.1038/s41598-019-40740-1)
Supplement: Supplementary file 1 — Supporting material [file 41598_2019_40740_MOESM1_ESM.pdf]

Supplementary Information

Tsunamigenic potential of crustal faults and subduction zones in the Mediterranean

by

Patrizio Petricca* and Andrey Babeyko

GFZ German Research Centre for Geosciences, Potsdam, Germany

Corresponding author:
Patrizio Petricca *Now at Dipartimento di Scienze della Terra. Università Sapienza. Roma. Italy
e-mail:patrizio.petricca@uniroma1.it

1. Tsunamigenic potential: a coastal perspective.

We present here the tsunamigenic potential of the source database by simple counting the number of scenarios (individual sources) producing different threshold levels at POIs. Note that while presenting the absolute numbers (counts), we still effectively provide the relative information since the total number of our scenarios (about 20000) depends on the adopted discretization of CSS and SUBD interfaces. Moreover, these counts have no direct relation to the tsunami impact probability because we do not assign likelihoods to our sources. Nevertheless, plotting all scenarios together gives an idea about general tsunami hazard distribution in the whole region.

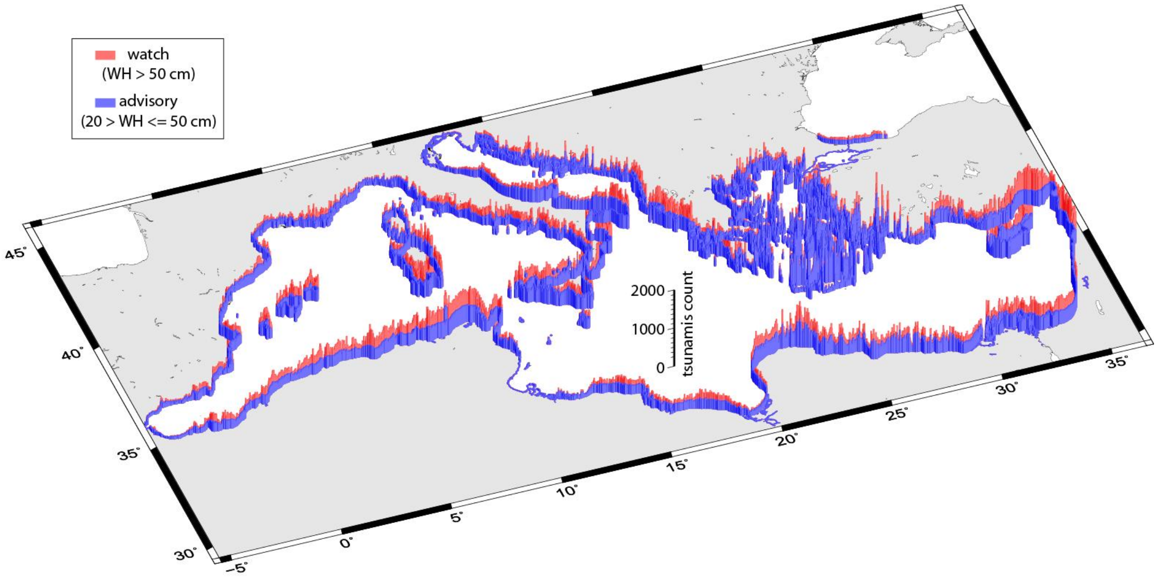

Figure S1 - Number of total advisory (blue) and watch (red) tsunami occurrences due to the whole set of CSS. Red bars are stacked atop of the blue ones.

Figure S1 presents such compilation for CSS sources only. From this compilation we can see that almost the whole Mediterranean coast is subjected to potential tsunamis at the ‘advisory’ (i.e., wave height exceeding 20 cm and under 50 cm; blue bars) and ‘watch’ (i.e., wave height exceeding 50 cm; red bars) alert levels. The largest number of occurrences is close to the Hellenic arc. The total number of events is high along north-African and Italian coasts.

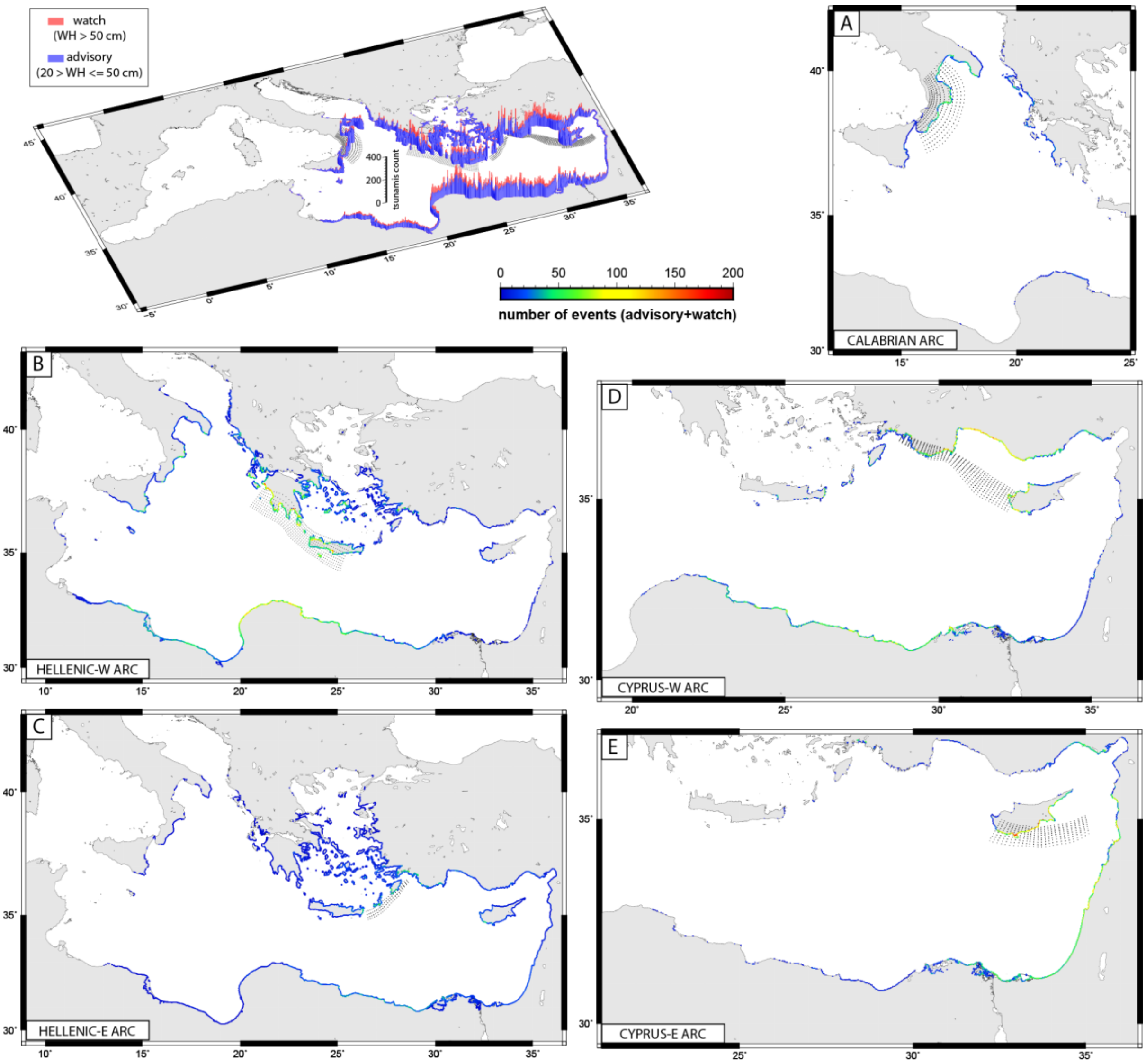

Figure S2 - Number of total advisory (blue) and watch (red) tsunami occurrences due to the whole set of SUBD. De-aggregation of events counting produced by each subduction interface is also given in panels for A) Calabrian arc, B) Hellenic West and C) East arcs, D) Cyprus West and E) East arcs.

Noteworthy that gaps and peaks in bar heights characterize the East-Tunisia-Libya and South Turkey coasts, respectively. A relatively low number of ‘watch’ events was predicted for Spain and France. Among subduction zones (Figure S2), events produced at the Calabrian Arc can be considered as more local (Figure S2a). Relatively high number of ‘advisory’ or

‘watch’ level tsunamis is recorded along the Italian coast, but, nevertheless, this amount never exceeds 50 occurrences. Few occurrences affect the eastern Greek and Libyan coasts at the longitude of Benghazi.

The Hellenic West and East Arcs are the most effective tsunamigenic sources in the region due to their greater size and higher maximal assigned magnitude (Mw_{max} 8.4). Considering results for the West Hellenic Arc (Figure S2b), peaks of occurrences (> 100) are attributed to the nearby coasts of Peloponnese and Crete and, in a more regional perspective, to the Benghazi promontory in Libya. A relative large number of occurrences is also recorded in the Aegean Sea as well as along Sicily and Calabria in South Italy. Few events reach the easternmost Mediterranean coasts of Syria and Lebanon due to the barrier represented by Cyprus Island and, south of it, the Eratosthenes Seamount (see Figure 1 of the main text). With the exception of some coasts suffering more than 75-100 events in the very near islands, the East Hellenic Arc affects the Eastern Mediterranean more evenly and with less occurrences (Figure S2c). Only few green spots (i.e., over 75 events) can be recognized at the Egyptian and West Cyprus coasts.

Although West and East segments of the Cyprus Arc with Mw_{max} 7.6 are thought to be less tsunamigenic compared to the Hellenic subduction (Mw_{max} 8.4), they still keep the high possible impact at the easternmost coast of Mediterranean (Figure S2d-e). Large areas of Egypt, South Turkey, Mid-East (Syria, Lebanon and Israel) and Cyprus recorded between 70-200 virtual events above the ‘advisory’ level. Notable that tsunamis triggered at the Cyprus Arc do not propagate into the Aegean Sea as well as west to Benghazi.

2. Comparison with results by Necmioglu and Ozel (2015)[11] for the East Mediterranean.

Recently, Necmioglu and Ozel[11] published an extensive databank of synthetic tsunami scenarios in the East Mediterranean with earthquake sources distributed by location, depth (‘shallow’ with upper edge at 5 km and ‘deep’ with that at 40 km), magnitude and focal mechanism in accord with regional tectonics and seismicity. To compare our results with this dataset, we have post-processed their data and extracted minimum tsunamigenic magnitudes according to our definition (see main text). Figure S3 presents minimum magnitudes for ‘advisory’ (upper-left panel) and ‘regional watch’ (upper-right panel) tsunami alerts.

Minimum tsunamigenic magnitudes demonstrate the same tendency to increase from ‘advisory’ to ‘regional-watch’ alert levels. However, in comparison to our simulation results, these simulations generally show lower minimum tsunamigenic magnitudes, especially for

the ‘regional watch’ level: compare Figure S3 (upper-left) to Figure 3 (lower panel) of the main text. Apparently, this difference is attributed to the methodological issues of the both studies. Necmioglu and Ozel[11] scenario database is aimed for the tsunami early warning. At each gridded geographical location, the database contains several scenarios varying by magnitude and depth; the latter can be either 5, or 40 km (top edge of a fault).

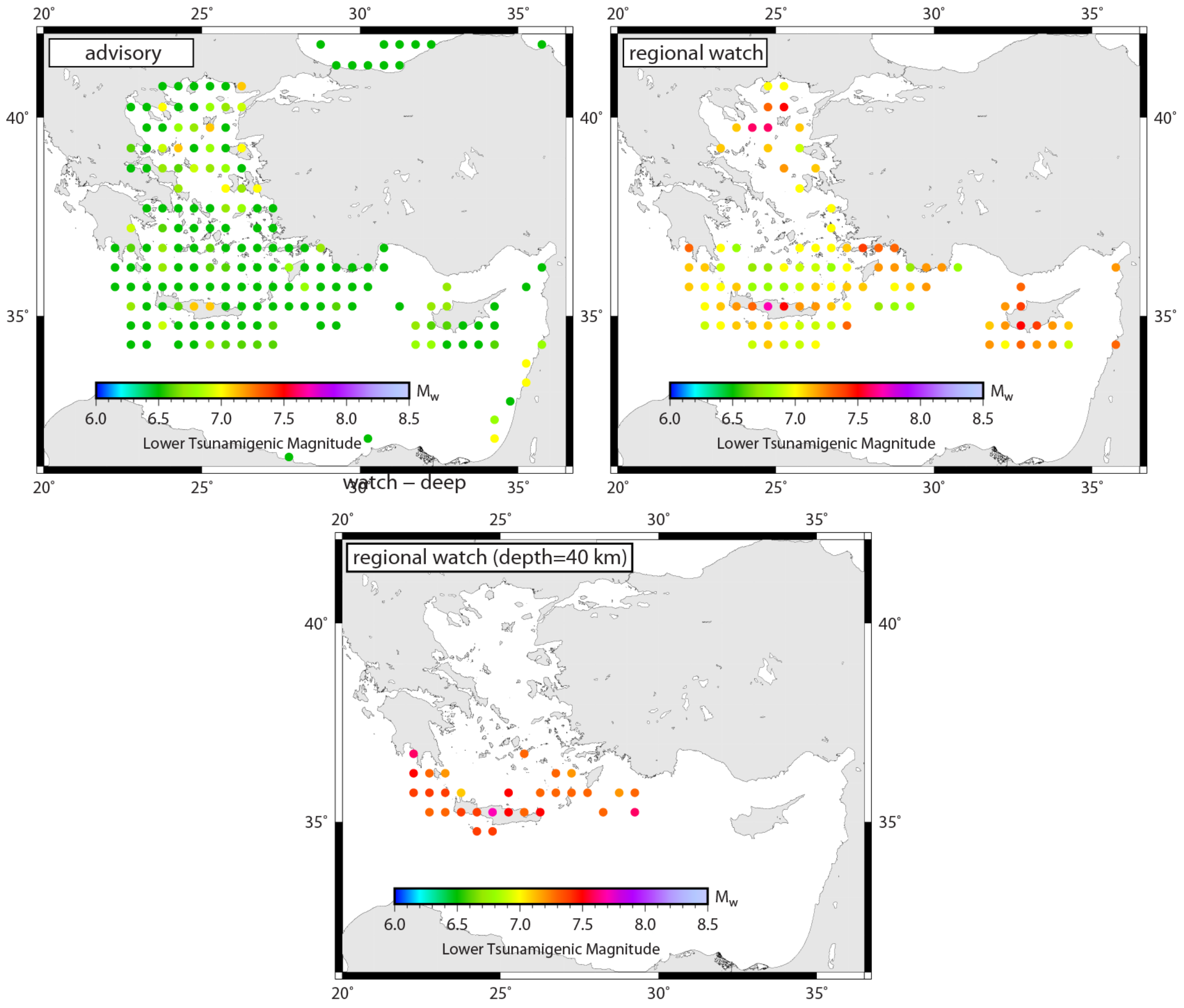

Figure S3 - Minimum tsunamigenic magnitude (Mw_{min}) of gridded sources from the KOERI compilation (Necmioglu and Ozel. 2015) necessary to produce *advisory* and regional *watch* tsunami alerts. Upper-left panel- at any source-to-target distance; upper-right and lower panels- at distances >100 km. Lower panel: deep sources only.

With this configuration, simulated hazard will be dominated by shallowest sources at each gridded location. On another hand, in our simulation database, not every geographical location within CSS or SUBD structures contains shallow events. Hypocentre depths of individual sources follow dipping geometries of the corresponding structures. As a result, our scenario database has much smaller number of shallow events, and they are distributed very un-evenly in comparison with the Necmioglu and Ozel[11] gridded sources. For example, our

individual sources along the both Hellenic Arcs start from 18 km depth and deepen down. At the locations where we have shallow CSS sources, our Mw_{min} are in accord with Necmioglu and Ozel[11] results: compare CSS sources near Cyprus (Figure 3, lower panel) with gridded sources at the same location on Figure S3. In contrast, there are no shallow CSS sources nearby the East-Hellenic Arc, and the up-dip boundary of the NW-dipping seismogenic zone lies already at 18 km. As a result, our generally deeper sources require larger magnitudes to trigger the same tsunami alert level than the shallow sources from Necmioglu and Ozel (2015) do. If we neglect their shallow (top edge at 5 km) and consider only deep sources (top edge at 40 km), then Mw_{min} along the Hellenic Arcs become similar in the two datasets: compare Figure S3 (lower panel) with Figure 3 (lower panel).

Another methodological difference lies in the post-processing. As described above in the main text. In order to 'label' a source as 'tsunamigenic', we counted at least 10 POIs over the corresponding threshold. This was done to remove possible outliers since our POIs (>20 000) were distributed automatically and not checked for occasional mal-positioning. Experiments with less minimal counts always resulted in lower Mw_{min} (sometimes. maybe due to outliers). By post-processing Necmioglu and Ozel (2015) scenario database, we did not apply this 'minimum-count' criterion: the source was marked as 'tsunamigenic' if the wave height overridden an alert level threshold at any single POI.

3. Assigning grid nodes to test-sites

When computing tsunami threads for the selected test-sites via de-aggregation (see main text, Figure 4), we identified each test site through a cloud of grid nodes rather than assigning a single node to it. As noted in the main text, it was done to suppress possible outliers and to obtain an averaged, smoothed and, thus, more representative, tsunami impact over the community coastline. Effective wave height for a test-site was calculated as an average wave height over the cloud of grid nodes assigned to the test-site. Since the average value is always smaller than the maximum over population, Mw_{min} based on community-averaged wave height should be a conservative estimate for the community. Figure S4 demonstrates assignment of computational grid nodes to test-sites: selection of points generally follows geographic location and extent of corresponding communities.

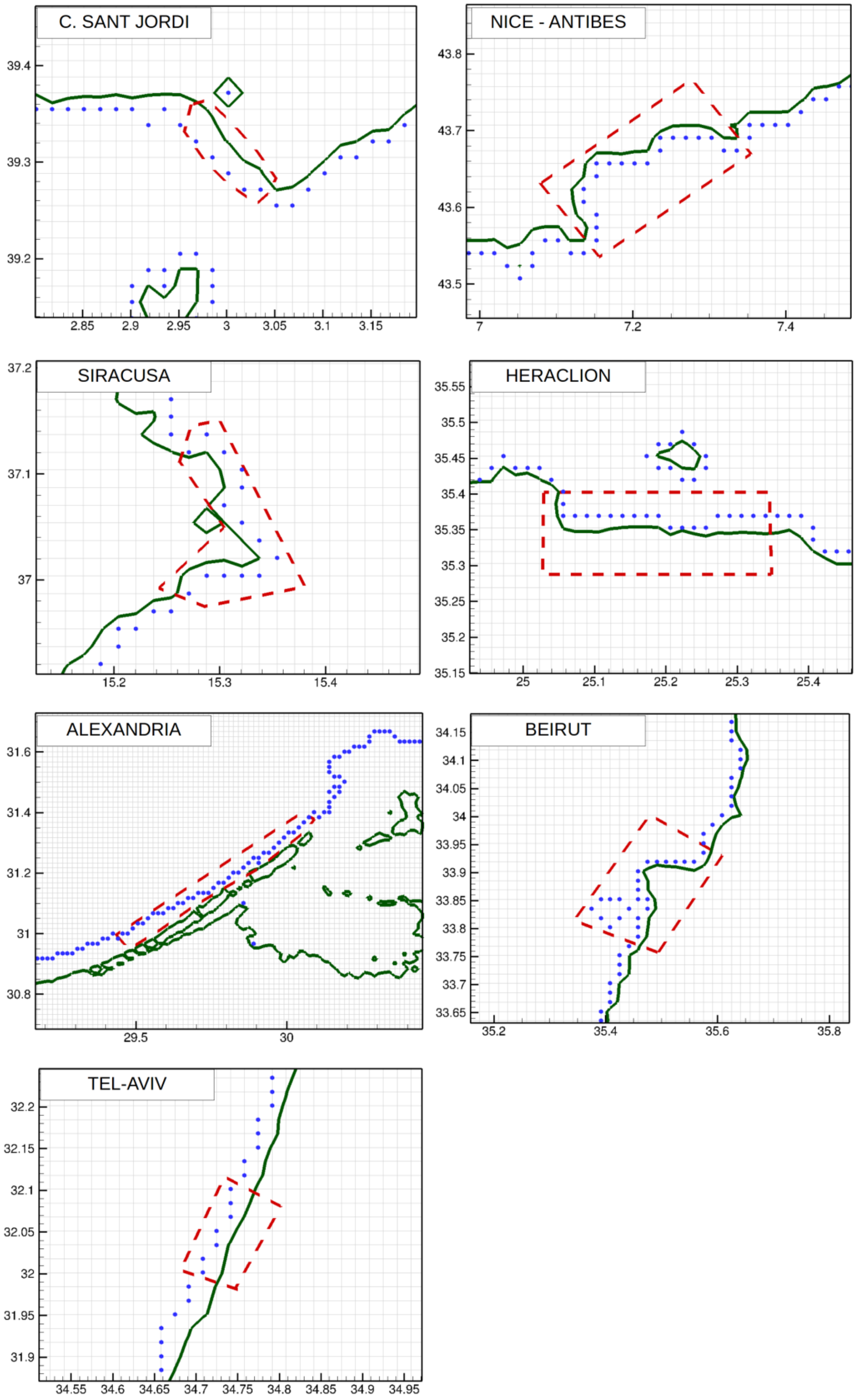

Figure S4 – Grid nodes assignment to the test-sites. Effective wave height for a test-site will be computed as average over coastal POIs (blue dots) belonging to the site (red dashed contours).

4. Sensitivity tests

4.1 Sensitivity to propagation model: placement of the reflecting wall and POIs

We simulate tsunami propagation in a linear long-wave approximation with full-reflection at the water-land boundary; see *Methods* section in the main text for more detailed description.

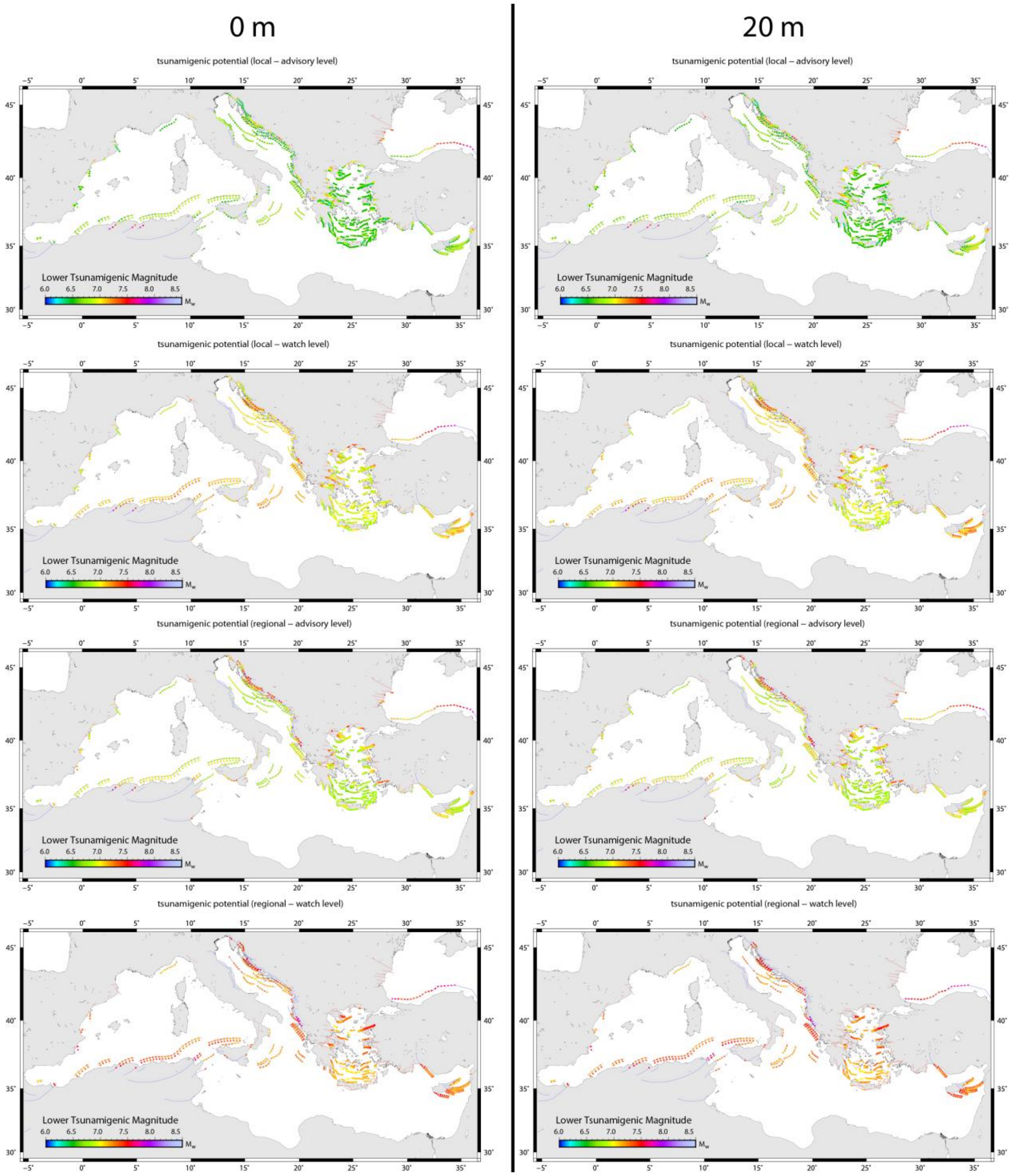

Figure S5 – Sensitivity study: Propagation model: Placement of the reflecting wall (and coastal POIs). Left side- reflecting wall at 0-meter isobaths; right side- at 20-m isobaths.

Coastal points-of-interest (POIs) where we do measure wave heights are distributed along the reflecting coastal wall. Here we test sensitivity of our metric – minimum tsunamigenic magnitude Mw_{min} – to the positioning of the wall. In the first case (Figure S5, left), we set the wall along the zero-meter isobaths, i.e., directly along the coast as given by the topo-bathymetric grid (in our case: Gebco_08). In the second case (Figure S5, right), for which we actually present all our results in this paper, the wall was placed along the 20-meter isobaths. Moving the reflecting wall into deeper water is a usual approach when using linear tsunami simulations: it restricts computations to the validity domain of linear approximation and also allows avoiding small-scale coastal features like shallow bays which would require much higher spatial resolution. Comparison of the two models (Figure S5) demonstrates no visible difference between the two propagation models.

4.2 Sensitivity to source parameters

Composite seismic sources (CSS) are described by their geographical shape and representative focal parameters including strike, dip and rake angles. For the most CSS's, these parameters are not unique but allow some variations. To check influence of the possible variations on our results, we conducted a sensitivity test based on one selected CSS structure at the North-Algerian margin (DZSC001).

This CSS hosts 10 individual source locations: 5 at a shallow level and 5 at a deeper level (see Figure S6). For each location and magnitude (6.3 – 8.0), we have systematically repeated our Mw_{min} analysis by disturbing reference rupture parameters. In particular:

- Each individual source location was shifted 30 km NW and 30 km SE (see Fig. S6);
- Strike, dip, and rake angles were varied by +/-10 degrees.

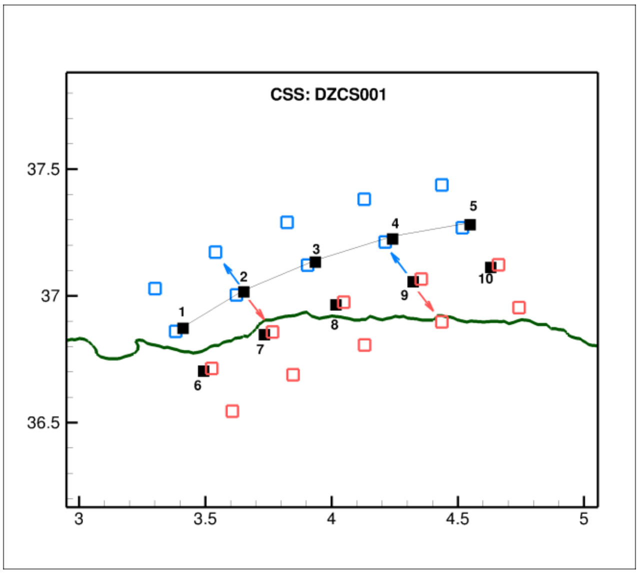

Figure S6 – Checking sensitivity to lon/lat positions of individual sources. Here- compositional seismic source DZCS001 (North Algerian coast, host structure of the 2003 Bourmedes M6.9 earthquake). Black boxes– original positions of shallow (1-5) and deep (6-10) sources. Blue dots- test positions shifted 30 km NW, red dots- shifted 30 km SE.

Tables ST1 and ST2 summarize the variability of the resulting Mw_{min} metric in respect to the given variations of source parameters. Table ST1 presents variation range of Mw_{min} at the *'advisory'* alert level, while Table ST2 refers to the *'regional watch'* alert level. Minimum tsunamigenic magnitude does not vary markedly due to the variations of focal parameters: typical variation is 0.1 with maximum value of 0.2 (fifth column of the Tables). Additional incorporation of the geographical shift for 30 km has more pronounced effect: see the last column of the both Tables. Mw_{min} variations are significantly larger for individual sources positioned close to the shoreline- compare, e.g., position 6 against 5. The reason is obvious: if source is located close to the shoreline, volume of the uplifted water drastically depends on the exact source position. This is clearly represented in Figure S7 where the relative error is shown. Regardless of the considered warning level, the variation due to shift in the source position is higher but always less than 10% moving the source to the north, i.e. toward the open Sea. Moving the source on-shore (to the south) produces variations in the obtained Mw_{min} less than 5%.

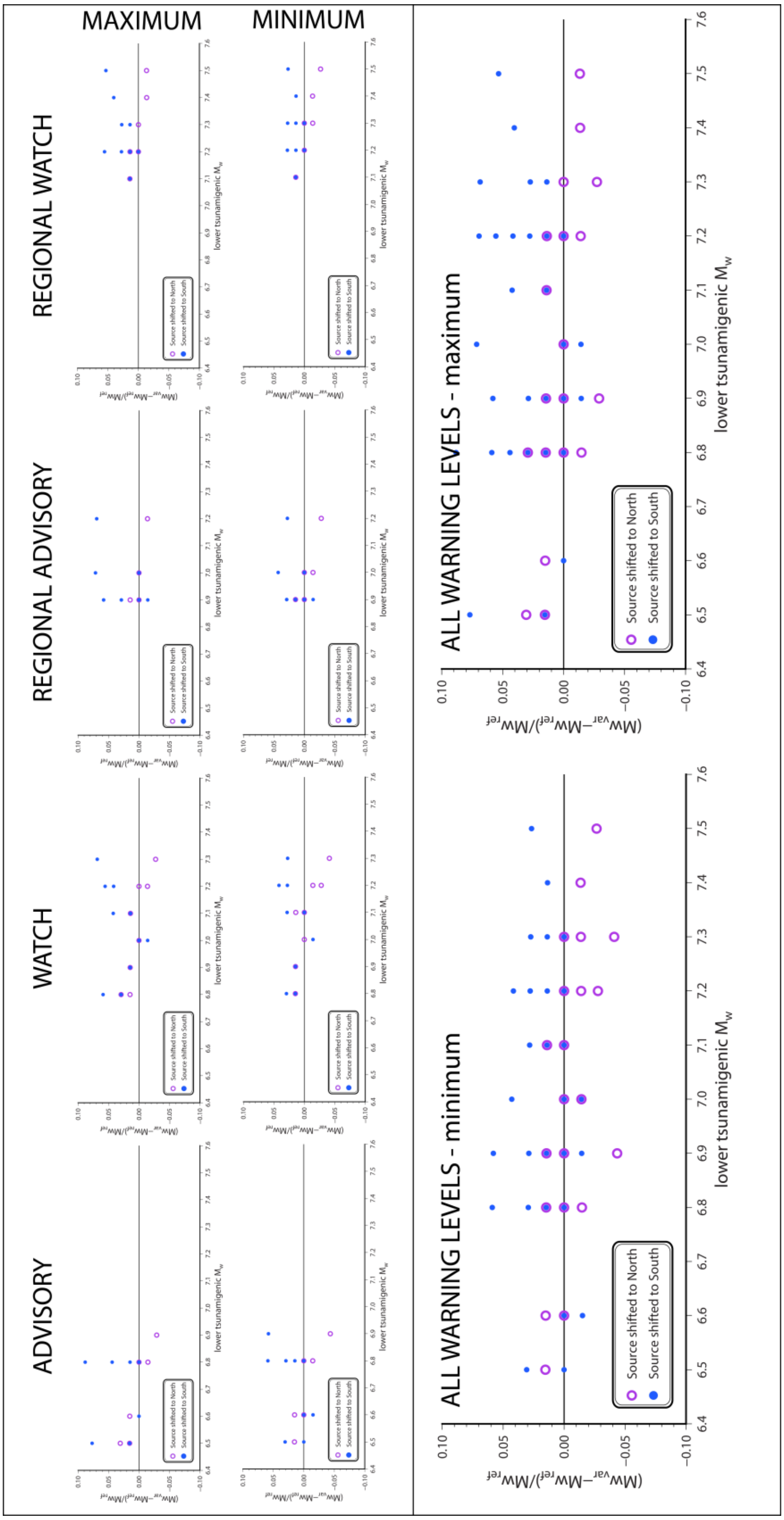

Figure S7 – Checking sensitivity to lon/lat positions of individual sources. Here- compositional seismic source DZCS001 (North Algerian coast, host structure of the 2003 Bourmedes M6.9 earthquake). Black line represents the original position (Black boxes in Figure S6). Purple circles give relative error obtained at test positions shifted 30 km NW; blue dots give relative error at test positions shifted 30 km SE. Notice that the variation due to shift in the source position is always less than 10%.

Table ST1 – Sensitivity of the Mw_{min} (*'advisory'* alert) to variations in source positions and focal parameters

posID	lon	lat	Reference Mw_{min}	Variations by strike, dip and rake	… plus position shift
1	3.41259	36.8704	6.5	6.5 ÷ 6.5	6.5 ÷ 7.0
2	3.65318	37.0147	6.6	6.5 ÷ 6.6	6.5 ÷ 6.7
3	3.93598	37.1317	6.6	6.6 ÷ 6.6	6.5 ÷ 6.7
4	4.24246	37.2237	6.6	6.6 ÷ 6.6	6.5 ÷ 6.7
5	4.54894	37.2799	6.7	6.6 ÷ 6.7	6.6 ÷ 6.7
6	3.49432	36.7018	7	6.9 ÷ 7.1	6.6 ÷ 7.6
7	3.73491	36.8462	6.9	6.8 ÷ 6.9	6.7 ÷ 7.4
8	4.01771	36.9632	6.9	6.8 ÷ 6.9	6.8 ÷ 7.1
9	4.32419	37.0551	6.8	6.8 ÷ 6.8	6.8 ÷ 6.9
10	4.63066	37.1113	6.8	6.8 ÷ 6.8	6.8 ÷ 6.8

Table ST2 – Sensitivity of the Mw_{min} (*'regional watch'* alert) to variations in source positions and focal parameters

posID	lon	lat	Mw_{min}	Variations by strike, dip and rake	… plus position shift
1	3.41259	36.8704	7.3	7.2 ÷ 7.3	7.2 ÷ 7.6
2	3.65318	37.0147	7.3	7.2 ÷ 7.3	7.2 ÷ 7.4
3	3.93598	37.1317	7.2	7.1 ÷ 7.2	7.1 ÷ 7.2
4	4.24246	37.2237	7.3	7.2 ÷ 7.3	7.2 ÷ 7.3
5	4.54894	37.2799	7.2	7.2 ÷ 7.2	7.2 ÷ 7.3
6	3.49432	36.7018	7.6	7.5 ÷ 7.6	7.3 ÷ 7.9
7	3.73491	36.8462	7.4	7.4 ÷ 7.4	7.3 ÷ 7.7
8	4.01771	36.9632	7.3	7.3 ÷ 7.3	7.2 ÷ 7.5
9	4.32419	37.0551	7.4	7.3 ÷ 7.4	7.3 ÷ 7.4
10	4.63066	37.1113	7.4	7.3 ÷ 7.4	7.3 ÷ 7.4

4.3 Sensitivity to the POI 'count threshold'

As explained in the main text, we employed a group POI evaluation to declare a particular individual source as tsunamigenic or not. A source was declared as tsunamigenic if it was able to trigger tsunami over given wave height (wave-height threshold) at more than given number of POIs (POI count threshold). Such group evaluation plays a role of filtering of possible outliers, but, on another hand, tends to increase the Mw_{min} estimate (that is why we consider our resulting minimum tsunamigenic magnitudes as rather conservative estimates). In order to evaluate the sensitivity of Mw_{min} to the count threshold, we have made a detailed systematic analysis taking the above mentioned CSS DZCS001 as an example. Table ST3

and ST4 present sensitivities of the '*advisory*' and '*regional watch*' Mw_{min}'s to the count thresholds, respectively.

Table ST3 – Sensitivity of the Mw_{min} (*'advisory'* alert) to the POI count threshold. Individual sources 1-10 belong to CSS DZCS001 (see Tables ST1-2). Cnt=10 is a reference count threshold used in this study.

posID	lon	lat	Mw_{min} @ cnt = 3	Mw_{min} @ cnt = 5	Mw_{min} @ cnt = 10	Mw_{min} @ cnt = 20	Mw_{min} @ cnt = 100
1	3.41259	36.8704	6.5	6.5	**6.5**	6.6	7.1
2	3.65318	37.0147	6.5	6.5	**6.6**	6.6	7
3	3.93598	37.1317	6.5	6.6	**6.6**	6.7	7
4	4.24246	37.2237	6.6	6.6	**6.6**	6.7	7
5	4.54894	37.2799	6.6	6.6	**6.7**	6.7	6.9
6	3.49432	36.7018	6.9	7	**7**	7.1	7.4
7	3.73491	36.8462	6.7	6.8	**6.9**	7	7.2
8	4.01771	36.9632	6.7	6.8	**6.9**	6.9	7.1
9	4.32419	37.0551	6.8	6.8	**6.8**	6.9	7.1
10	4.63066	37.1113	6.7	6.8	**6.8**	6.9	7.1

Table ST4 – Sensitivity of the Mw_{min} (*'regional watch'* alert) to the POI count threshold. Individual sources 1-10 belong to CSS DZCS001 (see Tables ST1-2). Cnt=10 is a reference count threshold used in this study.

posID	lon	lat	Mw_{min} @ cnt = 3	Mw_{min} @ cnt = 5	Mw_{min} @ cnt = 10	Mw_{min} @ cnt = 20	Mw_{min} @ cnt = 100
1	3.41259	36.8704	7.3	7.3	**7.3**	7.4	7.5
2	3.65318	37.0147	7.2	7.2	**7.3**	7.3	7.4
3	3.93598	37.1317	7.2	7.2	**7.2**	7.3	7.4
4	4.24246	37.2237	7.2	7.2	**7.3**	7.3	7.4
5	4.54894	37.2799	7.1	7.2	**7.2**	7.3	7.4
6	3.49432	36.7018	7.6	7.6	**7.6**	7.6	7.8
7	3.73491	36.8462	7.4	7.4	**7.4**	7.5	7.6
8	4.01771	36.9632	7.2	7.3	**7.3**	7.4	7.5
9	4.32419	37.0551	7.3	7.3	**7.4**	7.4	7.5
10	4.63066	37.1113	7.3	7.3	**7.4**	7.4	7.5

Please note, that cnt=10 is our reference count threshold accepted in this study. At a bathymetric grid with 1 arc minute resolution, this number of grid nodes roughly corresponds to 20 km of coastline. Inspection of the Tables ST3-4 justifies our choice of the count threshold: Mw_{min} values remain stable (within 0.2 magnitude unit) between cnt=3 and cnt=20. Further increase of the count threshold to cnt=100 leads to markedly higher Mw_{min} values, especially for the case of the '*advisory*' alert (Table ST4).
